# Risk of dialysis initiation within 20 years after living kidney donation in Japan: a period estimate from the national dialysis and transplant registries

**DOI:** 10.3389/ti.2026.17852

**Published:** 2026-10-07

**Authors:** Kenta Torigoe, Tomohisa Tsuyuki, Mayu Iwata, Emiko Otsuka, Kiyokazu Tsuji, Ayuko Yamashita, Miki Torigoe, Mineaki Kitamura, Kumiko Muta, Ryoichi Imamura, Tomoya Nishino

**Affiliations:** 1 Department of Nephrology, Nagasaki University Hospital, Nagasaki, Japan; 2 Department of Urology, Nagasaki University Graduate School of Biomedical Sciences, Nagasaki, Japan

**Keywords:** dialysis, Japan, kidney failure, living kidney donor, long-term outcome, registry

Dear Editors,

Long-term renal prognosis of living kidney donors underpins informed consent. National estimates exist for the United States [1, 2] and Norway [3], with age-specific projections [4]. Japanese donors differ: over 40% are spouses (unrelated) and half are aged ≥60 at donation [5, 6]. Japanese evidence comes from single-centre series [7, 8], and national follow-up loses about 30% of donors by 11 years [5]. Since 2019 the Japanese Renal Data Registry (JRDR) has recorded a “history of kidney donation as a donor” in incident dialysis patients. We combined this item with the national living-donor registry to estimate, without individual linkage, the 20-year risk of dialysis initiation.

Among 222,117 patients in the JRDR patient survey who started dialysis in 2019–2024 (94% of the 236,991 incident patients counted by the facility survey), donor history was recorded in 140,779 (63.4%), of whom 94 had donated a kidney; the interval since donation was known for 76 (unknown in 18) and shorter than 240 months in 37. All JRDR figures are aggregate cross-tabulations obtained through the Web-based Analysis of Dialysis Data Archives (WADDA) system of the Japanese Society for Dialysis Therapy. The denominator comprised 32,015 living-donor transplants in 1999–2024 (annual counts [5, 6]; age and sex distribution [6]), each donation-year cohort contributing donor-years to the 0–9 and 10–19-year intervals in 2019–2024 (period approach). Cause-specific hazards on death-censored donor-years (complete life tables [9]) were converted into cumulative incidence with death as a competing risk (Aalen–Johansen; current donor age mix). The primary estimate allocated interval-unknown cases proportionally (×1.24) and imputed cases among patients without recorded donor status, including non-responding facilities, at the same prevalence (×1.68); the unadjusted and the death-censored (net) estimates bound it below and above. Age at donation, recorded since 2021, was analysed for 2021–2024 (exploratory; unknown in eight of 66 cases, re-allocated). Standardized incidence ratios (SIR) applied year-, sex- and age-specific JRDR incidence to death-censored donor-years by attained age. 95% Monte-Carlo intervals resampled counts, the allocation of interval-unknown cases and the missing-data factor, not denominators.

The adjusted 20-year cumulative incidence of dialysis initiation was 1.1% (95% interval 0.8–1.5) (Figure 1A; Supplementary Table S1a); unadjusted 0.5% (0.4–0.7), net 1.3% (1.0–1.8). The 20-year risk was 0.8% (0.3–1.3) for donation before 60 and 1.6% (0.9–2.5) at ≥60 (Figure 1B; Supplementary Table S1c). The adjusted SIR versus the general population was 1.23 (0.86–1.69), 0.59 (0.41–0.81) if no unrecorded patient is a donor, and similar by sex (Supplementary Tables S1d, S2). Of the 76 initiations with a known interval, 39 occurred ≥20 years after donation; extending denominators to 1971 [5] gave 2.2% at 30 years (Supplementary Table S3).

**FIGURE 1:**
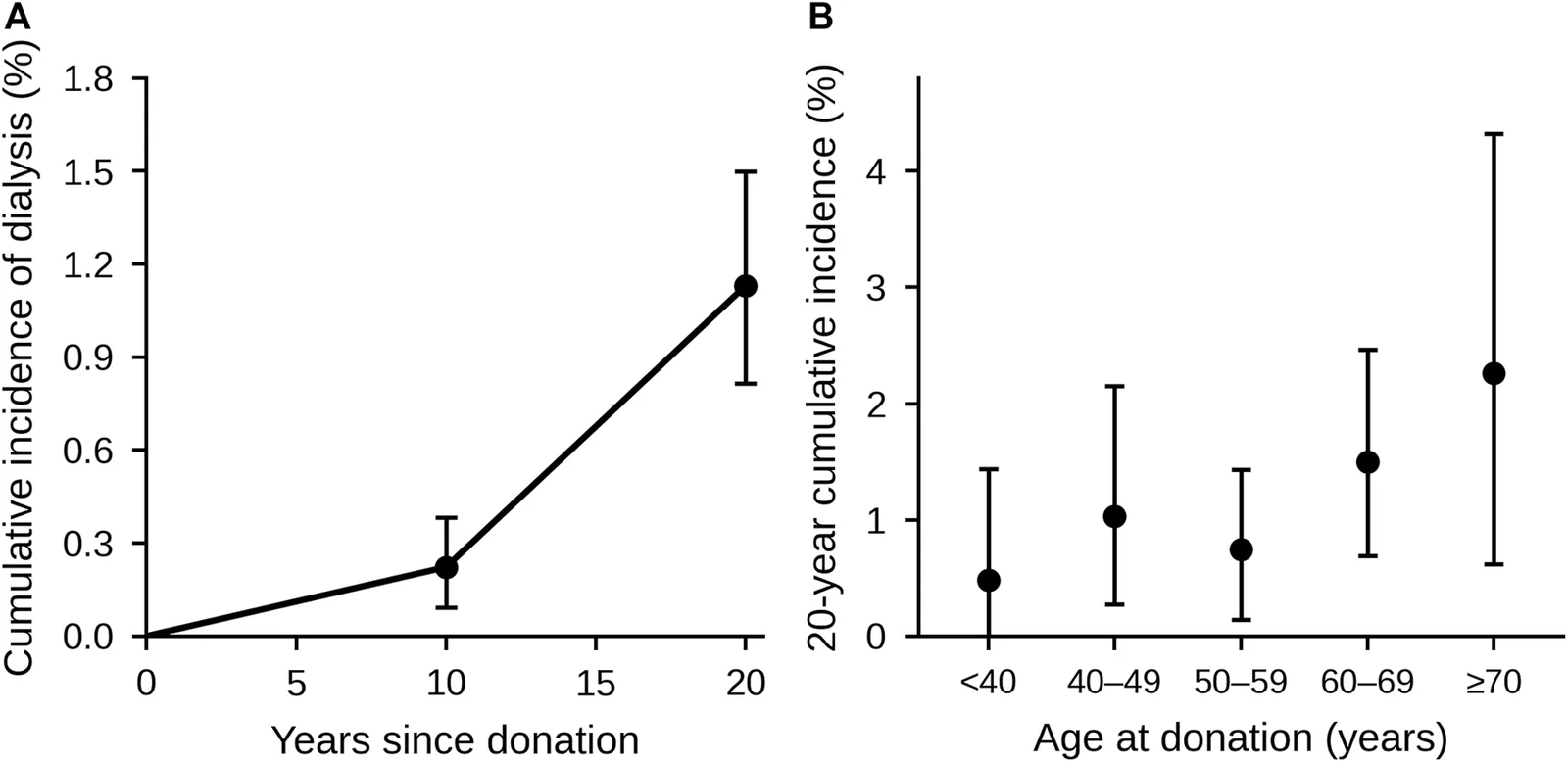
Cumulative incidence of dialysis initiation after living kidney donation in Japan (JRDR 2019–2024), with death as a competing risk and adjusted for interval-unknown cases (×1.24) and unrecorded donor status (×1.68). **(A)** All donors: estimates at 10 and 20 years (37 initiations within 20 years; donations 1999–2024). **(B)** Twenty-year estimates by age at donation (JRDR 2021–2024, 27 initiations; exploratory). Bars, 95% Monte-Carlo intervals (random error only; systematic uncertainty is bounded in Supplementary Table S4c–e). Counts, donor-years and rates in Supplementary Table S1.

These are the first nationwide estimates for Japan. In plain terms, roughly one donor in a hundred starts dialysis within 20 years of donation (range about one in 200 to one in 50), about one in 60 after donation at ≥60, and about 2% (roughly one in 50) by 30 years, because half of the recorded initiations came later than 20 years (Supplementary Table S3; exploratory, as denominators before 1999 cannot be verified). The estimate rests on assumptions that cannot be verified without linkage, so we bounded them (Supplementary Table S4c–j): prevalence of donor history among unrecorded patients 0–1 times that among recorded (recording completeness varied without association with prevalence, and missingness was block-wise; Supplementary Table S4); item sensitivity ≥0.6, weakly constrained by the transplant-society follow-up and the transplant-history item; and positive predictive value ≥ 0.75 (26% of “donors” in the year-end survey had a recorded transplant). The resulting range, approximately 0.5%–2%, is not a confidence interval, and values above 2% cannot be excluded if sensitivity is below 0.6 (2.2% at 0.5). Comparison with the general population is a weak yardstick for two reasons. Donors are selected for good health, so their risk should lie well below that of the general population, which includes people with diabetes and kidney disease; US donors have 0.3 times its lifetime risk [1], so a ratio near 1 would already imply excess risk. And because 41% of incident patients had no record of donor status, the ratio depends on the accuracy of that record: the SIR of 1.2 falls below 1 at a positive predictive value of 0.8 (Supplementary Table S4k). These data therefore cannot tell whether Japanese donors carry an excess risk or whether selection is less stringent (nearly a quarter of recent Japanese donors have hypertension, most of them treated [6]); the absolute risk, not the ratio, is the figure to use. The net 1.3% is 2.7 times the US 20-year figure (0.49%, death-censored, median age 41, counting transplantation and wait-listing [2]); for donation before 60 the ratio is 1.7 (1.4 at a positive predictive value of 0.8; Supplementary Table S1c), so age structure explains much of the gap, although it widens again against white (0.38%) or unrelated (0.25%) US donors [2]. Risk rises steeply with time and age [2, 4].

Several limitations apply. First, four in ten new dialysis patients had no record of donor status; the primary estimate assumes that donors are as frequent among them as among recorded patients, and the interval excludes this uncertainty. Second, misclassification runs both ways: 6 of the 94 had a recorded kidney transplant and 2 an incompatible primary disease (Supplementary Table S5; excluding these, 1.0%); but the registry has no code for kidney failure in a solitary kidney, so some true donor cases fall in “other”, where donor history was 14 times as frequent as in diabetic nephropathy, and a tumour in the remaining kidney (3 cases) is itself a route to dialysis; excluding all 24 over-excludes (0.8%; Supplementary Table S4j). Donors recorded as non-donors are undetectable. Third, the registry counts only dialysis: donors who received a pre-emptive transplant, died before dialysis or chose conservative management, all frequent at advanced age, are missed, understating risk after donation at ≥60; complete registry coverage and negligible emigration are assumed. Fourth, today’s donors are older than those whose second decade we observed (2000–2014 donations; Supplementary Table S6); standardizing 2021–2024 age-specific estimates to the current age mix gives 1.3% (Supplementary Table S1c), so the primary estimate probably understates risk for current older donors. Fifth, the estimate applies 2019–2024 rates to every interval since donation (a period estimate); rates did not differ by donation cohort (Supplementary Table S7).

In conclusion, approximately 0.5%–2% of living kidney donors in Japan (point estimate 1.1%, possibly higher if donor history is under-recorded) start dialysis within 20 years of donation, more often after donation at ≥60; this is the absolute risk after donation, not the risk attributable to it. Over half of recorded initiations occurred more than 20 years after donation, so the 20-year figure understates lifetime risk. For counselling, these figures do not by themselves change who may donate, but they give prospective Japanese donors a concrete, nationally derived number for consent, and they support follow-up of kidney function well beyond 20 years, particularly in older donors. Registry linkage [10] would give precise age- and sex-specific estimates.

## Data Availability

The data analyzed in this study is subject to the following licenses/restrictions: The aggregate data analysed in this study were obtained through the Web-based Analysis of Dialysis Data Archives (WADDA) system of the Japanese Renal Data Registry, Japanese Society for Dialysis Therapy, and from published reports of the Japan Society for Transplantation, the Japanese Society for Clinical Renal Transplantation, the Ministry of Health, Labour and Welfare and the Statistics Bureau of Japan. Under the WADDA system regulations, output tables may not be provided to nonmembers of the JSDT; JSDT members can reproduce the tabulations used in this study through the WADDA system (https://member.jsdt.or.jp/member/statistics). Requests to access these datasets should be directed to the corresponding author, Kenta Torigoe, ktorigoe@nagasaki-u.ac.jp.
