# Supplementary material for: Risk of dialysis initiation within 20 years after living kidney donation in Japan: a period estimate from the national dialysis and transplant registries
Source: Transpl Int. 2026 Oct 7;39:17852. doi: 10.3389/ti.2026.17852 (PMC13683231; doi:10.3389/ti.2026.17852)
Supplement: Supplementary file 1 [file Supplementaryfile1.docx]

**Supplementary Methods**

**1. Data sources**

Numerator: Japanese Renal Data Registry (JRDR) patient survey 2019–2024, obtained as aggregate cross-tabulations through the Web-based Analysis of Dialysis Data Archives (WADDA) system of the Japanese Society for Dialysis Therapy; no individual records were accessed. Donor history is the item ‘history of kidney donation as a donor’, recorded since the 2019 survey; age at donation since the 2021 survey. Facility-survey incident totals (all facilities) were taken from the annual JSDT reports.

Denominator: living-donor kidney transplants 1999–2024, annual counts from the Japan Society for Transplantation Fact Book and age and sex distribution from the Japanese Society for Clinical Renal Transplantation annual reports (not published for 1999 and 2001, taken from adjacent years; these contribute 0.5% and 2.4% of donor-years in the 10–19-year interval). Donations are placed at mid-year. Mortality: 19th–23rd complete life tables (2000–2020), linearly interpolated between tables and the 2020 table applied thereafter; donor age-group midpoints 35, 45, 55, 65 and 74 years. General-population incidence: JRDR incident counts by year, sex and 5-year age band over population estimates.

**2. Numerator and adjustments**

Cases are incident dialysis patients with recorded donor history and a donation-to-dialysis interval below 240 months. Interval-unknown cases (18 of 94) are allocated to the 0–9 and 10–19-year intervals in proportion to the known cases (×1.24). Cases among patients without recorded donor status are imputed at the prevalence among recorded patients (×1.68), covering both non-recording within the patient survey (×1.58) and patients at facilities not returning the patient questionnaire (×1.07; facility-survey total 236,991 vs 222,117). The unadjusted numerator (A) assumes no donors among unrecorded patients and is a lower bound.

**3. Donor-years (period approach)**

Each donation-year cohort contributes donor-years to the 0–9 and 10–19-year intervals for the part of 2019–2024 in which it falls in that interval. Death-censored donor-years reduce these by the survival of a donor of each age group and sex, chaining calendar-year-specific death probabilities. The second-decade interval is therefore populated by the 2000–2014 donation cohorts, which were younger at donation than current donors.

**4. Estimators**

Primary: adjusted competing-risk (Aalen–Johansen) cumulative incidence — cause-specific hazards (adjusted cases / death-censored donor-years, constant within each interval) applied to the survival of a donor of the current age mix (donations 2020–2024; 2020 life table). Scenarios: A, unadjusted; B, adjusted for interval-unknown cases and unrecorded donor status; C, B on death-censored donor-years (net risk); S, C excluding possibly misclassified cases. Reference estimator: the cumulative-hazard approximation 1 − exp(−Σ rate × width), on all donor-years (A, B) or on death-censored donor-years (C, S; the death-censored analogue of Kaplan–Meier estimates in donor cohorts). ‘Period’ refers throughout to the study design (rates of 2019–2024 applied across intervals since donation), not to an estimator. Direct standardization (Table S1c) weights the 2021–2024 age-specific competing-risk estimates by the current age mix; it gives 1.29% against 1.13% for the primary estimate, so the primary estimate probably understates risk for the current, older donor population.

**5. Monte-Carlo intervals**

Poisson resampling of observed counts; for adjusted estimates additionally Poisson–binomial resampling of the allocation of interval-unknown cases and a missing-data factor drawn uniformly over the range of stratified imputation ratios (0.998–1.033). Intervals cover random error only and exclude denominator uncertainty (annual counts, interpolated age distributions, life tables). Systematic uncertainty is bounded separately (Table S4c–e, k, l).

**6. Age at donation**

Exploratory; observation window 2021–2024, donors 2001–2024. Twenty-year age bands in the 2024 survey are split using the five-year distribution of the same cases. Adjusted cases = observed × 1.04 (interval unknown) × 1.14 (age at donation unknown in 8 of 66, re-allocated proportionally) × 1.68 (unrecorded donor status). The competing-risk estimate applies the age-specific hazards to the survival of a donor of that age; intervals resample counts only.

**7. Sex and standardized incidence ratios**

Sex: the observed male share of cases is compared with the share expected from donor-years by sex and the sex- and age-specific general-population incidence; the case-only comparison with the donor pool alone (exact binomial test; odds ratio from the Wilson interval) is shown for completeness. SIR: expected cases from year-, sex- and age-specific JRDR incidence 2019–2024 applied to death-censored donor-years at attained age; exact Poisson limits. The SIR scales linearly with the positive predictive value of the donor-history item (Table S4k).

**8. Validity checks and bias analysis**

Missing-at-random: recording completeness and donor-history prevalence by prefecture, facility type and survey year (Table S4a, b, f), joint missingness of the donor- and transplant-history items (S4i), and disease-stratified imputation (S4j). Sensitivity: transplant-society donor follow-up (Table S7d, e) and the internal consistency of the transplant-history item (S4g, h). Positive predictive value: transplant, tumour and ‘other’ primary diseases among recorded donors (S4j) and the year-end survey (S7b, c). Quantitative bias analysis over these three parameters gives the bounded range (S4c–e); its sensitivity to the PPV and to donor mortality is in S4k, l. Period assumption: donation-cohort and prevalent-patient checks (S7a–c).

**Table S1. Cumulative incidence of dialysis initiation within 20 years of living kidney donation in Japan (JRDR 2019–2024; period method)**

**(a) All donors, competing-risk (Aalen–Johansen) estimator, by scenario**

| **Scenario** | **Cases 0–9 y** | **Cases 10–19 y** | **Donor-years 0–9 y** | **Donor-years 10–19 y** | **Rate per 10,000, 0–9 y** | **Rate per 10,000, 10–19 y** | **10 y, % (95% MC)** | **20 y, % (95% MC)** |
| --- | --- | --- | --- | --- | --- | --- | --- | --- |
| A | 10.0 | 27.0 | 89,930 | 50,739 | 1.11 | 5.32 | 0.11 (0.04–0.18) | 0.54 (0.37–0.73) |
| B — primary estimate | 20.8 | 56.2 | 89,930 | 50,739 | 2.32 | 11.08 | 0.22 (0.09–0.38) | 1.13 (0.82–1.50) |

**(b) All donors, cumulative-hazard approximation, by scenario**

| **Scenario** | **Cases 0–9 y** | **Cases 10–19 y** | **Donor-years 0–9 y** | **Donor-years 10–19 y** | **Rate per 10,000, 0–9 y** | **Rate per 10,000, 10–19 y** | **10 y, % (95% MC)** | **20 y, % (95% MC)** |
| --- | --- | --- | --- | --- | --- | --- | --- | --- |
| A | 10.0 | 27.0 | 93,052 | 58,192 | 1.07 | 4.64 | 0.11 (0.04–0.18) | 0.57 (0.39–0.76) |
| B | 20.8 | 56.2 | 93,052 | 58,192 | 2.24 | 9.66 | 0.22 (0.09–0.38) | 1.18 (0.86–1.57) |
| C | 20.8 | 56.2 | 89,930 | 50,739 | 2.32 | 11.08 | 0.23 (0.10–0.40) | 1.33 (0.96–1.77) |
| S | 19.0 | 51.4 | 89,930 | 50,739 | 2.12 | 10.14 | 0.21 (0.09–0.36) | 1.22 (0.88–1.62) |

**(c) 20-year cumulative incidence by age at donation (2021–2024; exploratory)**

| **Age at donation, y** | **Cases <20 y** | **Donor-years 0–9 y** | **Donor-years 10–19 y** | **Net donor-years 0–9 y** | **Net donor-years 10–19 y** | **Approx. A** | **Approx. B** | **Approx. C (net)** | **Primary** |
| --- | --- | --- | --- | --- | --- | --- | --- | --- | --- |
| <40 | 1.0 | 3,466 | 4,116 | 3,454 | 4,051 | 0.24 (0.00–0.73) | 0.48 (0.00–1.44) | 0.49 (0.00–1.46) | 0.48 (0.00–1.44) |
| 40–49 | 3.8 | 10,311 | 7,302 | 10,232 | 7,042 | 0.52 (0.14–1.09) | 1.03 (0.27–2.15) | 1.06 (0.28–2.23) | 1.03 (0.27–2.15) |
| 50–59 | 5.2 | 18,286 | 13,810 | 17,974 | 12,662 | 0.38 (0.07–0.72) | 0.74 (0.14–1.43) | 0.81 (0.16–1.55) | 0.75 (0.14–1.43) |
| 60–69 | 10.7 | 21,693 | 12,952 | 20,765 | 10,588 | 0.77 (0.35–1.27) | 1.52 (0.70–2.51) | 1.83 (0.84–3.05) | 1.50 (0.69–2.47) |
| ≥70 | 6.3 | 9,467 | 3,562 | 8,634 | 2,026 | 1.19 (0.32–2.29) | 2.34 (0.63–4.50) | 3.64 (0.69–7.27) | 2.26 (0.62–4.32) |
| <60 (pooled) | 10.0 | 32,063 | 25,228 | 31,659 | 23,755 | 0.40 (0.16–0.67) | 0.78 (0.31–1.33) | 0.83 (0.33–1.41) | 0.78 (0.31–1.32) |
| ≥60 (pooled) | 17.0 | 31,160 | 16,514 | 29,399 | 12,614 | 0.88 (0.49–1.34) | 1.74 (0.96–2.64) | 2.20 (1.18–3.34) | 1.64 (0.90–2.48) |

**(d) Sex of cases within 20 years: comparison with general-population incidence**

|  | **Men** | **Women** | **Men, %** |
| --- | --- | --- | --- |
| Observed cases | 24 | 13 | 64.9 |
| Expected cases | 38.1 | 24.8 | 60.6 |
| Adjusted SIR (95% CI) | 1.31 (0.84–1.95) | 1.09 (0.58–1.87) |  |

**(e) Sex of cases within 20 years: case-only comparison with the donor pool**

| **Interval since donation** | **Men** | **Women** | **Women, %** | **Exact binomial p** | **Men:women odds ratio vs donor pool (95% CI)** |
| --- | --- | --- | --- | --- | --- |
| <10 y | 6 | 4 | 40.0 | 0.188 | 2.55 (0.77–8.42) |
| 10–19 y | 18 | 9 | 33.3 | 0.002 | 3.40 (1.56–7.43) |
| <20 y | 24 | 13 | 35.1 | <0.001 | 3.14 (1.62–6.10) |
| ≥20 y | 20 | 19 | 48.7 | 0.070 | 1.79 (0.97–3.32) |

Notes. MC, Monte-Carlo interval (random error only; Supplementary Methods 5); SIR, standardized incidence ratio. (a, b) Scenarios: A, unadjusted; B, adjusted (interval-unknown cases allocated, ×1.24; unrecorded donor status imputed, ×1.68); C, B on death-censored donor-years (net risk); S, C excluding 8 possibly misclassified cases (×0.915). Rates per 10,000 donor-years; cumulative incidence in %. Excluding all 24 ‘other’, tumour and transplant cases: 0.84% (Table S4j). (c) Approx., cumulative-hazard approximation; Net, death-censored donor-years; Primary, competing-risk estimator with adjusted cases (B). Age unknown in 8 of 66 cases, re-allocated (×1.14). Direct standardization to the current age mix: 1.29% (Supplementary Methods 4). (d, e) (d) Expected cases from year-, sex- and age-specific JRDR incidence on death-censored donor-years (Table S2); ratio of SIRs men : women 1.20 (0.61–2.36). (e) Exact binomial test against the pooled female share (63.0%); odds ratio from the Wilson interval; Fisher test <20 vs ≥20 y, p = 0.25. The case-only comparison ignores the male predominance of kidney failure.

**Table S2. Standardized incidence ratios (SIR) of dialysis initiation among living kidney donors versus the Japanese general population**

**(a) Within 20 years of donation (cohorts 1999–2024), by sex**

| **Sex** | **Observed** | **Expected** | **SIR, unadjusted (95% CI)** | **SIR, adjusted for unrecorded status (95% CI)** |
| --- | --- | --- | --- | --- |
| Men | 24 | 38.1 | 0.63 (0.40–0.94) | 1.31 (0.84–1.95) |
| Women | 13 | 24.8 | 0.52 (0.28–0.90) | 1.09 (0.58–1.87) |
| All | 37 | 62.9 | 0.59 (0.41–0.81) | 1.23 (0.86–1.69) |

**(b) All donation vintages (cohorts 1971–2024; all 94 cases; exploratory)**

| **Stratum** | **Observed** | **Expected** | **SIR, unadjusted (95% CI)** | **SIR, adjusted for unrecorded status (95% CI)** |
| --- | --- | --- | --- | --- |
| All | 94 | 85.9 | 1.09 (0.88–1.34) | 1.84 (1.49–2.26) |
| Men | 60 | 50.7 | 1.18 (0.90–1.52) | 1.99 (1.52–2.57) |
| Women | 34 | 35.2 | 0.97 (0.67–1.35) | 1.63 (1.13–2.27) |
| Age at initiation <60 y | 15 | 8.6 | 1.74 (0.98–2.88) | 2.94 (1.64–4.84) |
| Age at initiation 60–69 y | 8 | 18.7 | 0.43 (0.19–0.84) | 0.72 (0.31–1.42) |
| Age at initiation 70–79 y | 27 | 35.6 | 0.76 (0.50–1.10) | 1.28 (0.84–1.86) |
| Age at initiation ≥80 y | 44 | 23.0 | 1.91 (1.39–2.56) | 3.22 (2.34–4.32) |

Notes. Adjusted = unrecorded donor status imputed at the same prevalence (×1.68); exact Poisson limits. (a) Expected cases from year-, sex- and 5-year-age-specific JRDR incidence on death-censored donor-years by attained age. Ratio of SIRs men : women 1.20 (0.61–2.36). (b) Pre-1999 denominators as in Table S3; donor-years 2019–2024 216,372 (death-censored 175,382). Ratio of SIRs men : women 1.22 (0.80–1.87).

**Table S3. Extension beyond 20 years since donation (cohorts 1971–2024; exploratory; 12-month interval data, JRDR 2019–2024)**

**(a) Cases, donor-years and rates by interval since donation**

| **Interval since donation, y** | **0–9** | **10–19** | **20–29** | **30–39** | **40–49** |
| --- | --- | --- | --- | --- | --- |
| Observed cases (interval known) | 10 | 27 | 18 | 17 | 4 |
| All donor-years | 93,052 | 58,192 | 29,120 | 26,289 | 9,720 |
| Death-censored donor-years | 89,930 | 50,739 | 20,369 | 12,085 | 2,259 |
| Rate per 10,000 donor-years, scenario A | 1.07 | 4.64 | 6.18 | 6.47 | 4.12 |
| Rate per 10,000 donor-years, scenario B | 2.24 | 9.66 | 12.87 | 13.46 | 8.57 |
| Rate per 10,000 donor-years, scenario C | 2.32 | 11.08 | 18.40 | 29.29 | 36.87 |

**(b) Cumulative incidence by time since donation**

| **Scenario / estimator** | **10 y** | **20 y** | **30 y** | **40 y** | **50 y** |
| --- | --- | --- | --- | --- | --- |
| Approximation, A | 0.11 (0.04–0.18) | 0.57 (0.39–0.76) | 1.18 (0.86–1.53) | 1.82 (1.38–2.28) | 2.22 (1.65–2.85) |
| Approximation, B | 0.22 (0.09–0.38) | 1.18 (0.81–1.58) | 2.45 (1.78–3.17) | 3.75 (2.86–4.70) | 4.57 (3.41–5.84) |
| Approximation, C (net) | 0.23 (0.09–0.39) | 1.33 (0.91–1.78) | 3.13 (2.23–4.09) | 5.93 (4.39–7.57) | 9.33 (5.99–13.19) |
| Competing-risk, B (donor of the current age mix) | — | 1.13 (0.77–1.51) | 2.18 (1.59–2.80) | — | — |

Notes. MC, Monte-Carlo interval. Scenarios as in Table S1; rates per 10,000 donor-years; cumulative incidence in % (95% MC). Denominators before 1999: Fact Book annual counts (cumulative to 1971 assigned to 1971), JSCRT cumulative age distributions 1983–1990 and 1983–1994, female share of 2000; mortality before 2000 from the 2000 life table. Registry completeness before 1999 cannot be verified.

**Table S4. Missing donor status and misclassification: checks of the missing-at-random assumption and quantitative bias analysis (JRDR incident patients 2019–2024)**

**(a) Recording of donor status and prevalence of donor history by prefecture**

| **Prefecture** | **Donor history: yes** | **Donor history: no** | **Unknown** | **Not recorded** | **All incident patients** | **Recorded (yes + no)** | **Recording rate** |
| --- | --- | --- | --- | --- | --- | --- | --- |
| Hokkaido | 1 | 6,282 | 101 | 3,466 | 9,850 | 6,283 | 0.638 |
| Aomori | 1 | 1,875 | 1 | 863 | 2,740 | 1,876 | 0.685 |
| Iwate | 0 | 1,771 | 0 | 419 | 2,190 | 1,771 | 0.809 |
| Miyagi | 2 | 2,547 | 64 | 1,148 | 3,761 | 2,549 | 0.678 |
| Akita | 1 | 974 | 2 | 692 | 1,669 | 975 | 0.584 |
| Yamagata | 1 | 1,136 | 0 | 699 | 1,836 | 1,137 | 0.619 |
| Fukushima | 3 | 1,883 | 29 | 1,487 | 3,402 | 1,886 | 0.554 |
| Ibaraki | 0 | 3,399 | 11 | 2,021 | 5,431 | 3,399 | 0.626 |
| Tochigi | 2 | 2,409 | 25 | 1,601 | 4,037 | 2,411 | 0.597 |
| Gunma | 0 | 2,066 | 9 | 1,993 | 4,068 | 2,066 | 0.508 |
| Saitama | 2 | 7,501 | 60 | 5,495 | 13,058 | 7,503 | 0.575 |
| Chiba | 5 | 7,253 | 81 | 4,186 | 11,525 | 7,258 | 0.630 |
| Tokyo | 12 | 13,406 | 201 | 8,225 | 21,844 | 13,418 | 0.614 |
| Kanagawa | 9 | 9,587 | 119 | 4,268 | 13,983 | 9,596 | 0.686 |
| Niigata | 0 | 2,096 | 9 | 1,101 | 3,206 | 2,096 | 0.654 |
| Toyama | 1 | 1,166 | 0 | 567 | 1,734 | 1,167 | 0.673 |
| Ishikawa | 3 | 1,272 | 0 | 427 | 1,702 | 1,275 | 0.749 |
| Fukui | 3 | 763 | 13 | 446 | 1,225 | 766 | 0.625 |
| Yamanashi | 1 | 763 | 1 | 871 | 1,636 | 764 | 0.467 |
| Nagano | 0 | 2,034 | 5 | 1,353 | 3,392 | 2,034 | 0.600 |
| Gifu | 3 | 2,194 | 30 | 1,401 | 3,628 | 2,197 | 0.606 |
| Shizuoka | 2 | 4,553 | 23 | 2,846 | 7,424 | 4,555 | 0.614 |
| Aichi | 2 | 8,126 | 93 | 3,776 | 11,997 | 8,128 | 0.678 |
| Mie | 1 | 1,438 | 2 | 1,130 | 2,571 | 1,439 | 0.560 |
| Shiga | 0 | 1,757 | 23 | 645 | 2,425 | 1,757 | 0.725 |
| Kyoto | 0 | 2,319 | 125 | 1,430 | 3,874 | 2,319 | 0.599 |
| Osaka | 9 | 9,927 | 77 | 5,666 | 15,679 | 9,936 | 0.634 |
| Hyogo | 5 | 5,658 | 115 | 3,345 | 9,123 | 5,663 | 0.621 |
| Nara | 0 | 1,110 | 42 | 876 | 2,028 | 1,110 | 0.547 |
| Wakayama | 3 | 1,153 | 15 | 578 | 1,749 | 1,156 | 0.661 |
| Tottori | 1 | 747 | 0 | 395 | 1,143 | 748 | 0.654 |
| Shimane | 0 | 597 | 1 | 562 | 1,160 | 597 | 0.515 |
| Okayama | 0 | 2,699 | 70 | 913 | 3,682 | 2,699 | 0.733 |
| Hiroshima | 2 | 3,683 | 59 | 1,472 | 5,216 | 3,685 | 0.706 |
| Yamaguchi | 2 | 1,784 | 1 | 1,011 | 2,798 | 1,786 | 0.638 |
| Tokushima | 1 | 1,057 | 38 | 802 | 1,898 | 1,058 | 0.557 |
| Kagawa | 1 | 1,249 | 44 | 535 | 1,829 | 1,250 | 0.683 |
| Ehime | 3 | 1,550 | 0 | 1,019 | 2,572 | 1,553 | 0.604 |
| Kochi | 2 | 1,269 | 156 | 442 | 1,869 | 1,271 | 0.680 |
| Fukuoka | 1 | 5,992 | 82 | 3,595 | 9,670 | 5,993 | 0.620 |
| Saga | 1 | 1,079 | 21 | 383 | 1,484 | 1,080 | 0.728 |
| Nagasaki | 0 | 1,747 | 1 | 821 | 2,569 | 1,747 | 0.680 |
| Kumamoto | 3 | 2,202 | 32 | 727 | 2,964 | 2,205 | 0.744 |
| Oita | 2 | 1,469 | 42 | 780 | 2,293 | 1,471 | 0.642 |
| Miyazaki | 1 | 1,726 | 7 | 885 | 2,619 | 1,727 | 0.659 |
| Kagoshima | 2 | 1,813 | 23 | 1,165 | 3,003 | 1,815 | 0.604 |
| Okinawa | 0 | 1,601 | 4 | 938 | 2,543 | 1,601 | 0.630 |

**(b) Recording of donor status and prevalence of donor history by facility type**

| **Facility type** | **Donor history: yes** | **Recorded (yes + no)** | **All incident patients** | **Recording rate** | **Donors per 1,000 recorded patients (95% CI)** |
| --- | --- | --- | --- | --- | --- |
| National/public university | 0 | 1,721 | 3,491 | 0.493 | 0.00 (0.00–2.14) |
| Private university | 6 | 3,350 | 8,036 | 0.417 | 1.79 (0.66–3.90) |
| National Hospital Organization etc. | 0 | 997 | 1,899 | 0.525 | 0.00 (0.00–3.70) |
| Prefectural/municipal/NHI | 7 | 11,469 | 21,813 | 0.526 | 0.61 (0.25–1.26) |
| JCHO (former social insurance) etc. | 0 | 1,757 | 3,789 | 0.464 | 0.00 (0.00–2.10) |
| Koseiren (agricultural cooperative) etc. | 1 | 4,157 | 7,344 | 0.566 | 0.24 (0.01–1.34) |
| Other public | 7 | 5,915 | 13,144 | 0.450 | 1.18 (0.48–2.44) |
| Private general hospital | 4 | 4,443 | 7,823 | 0.568 | 0.90 (0.25–2.31) |
| Private hospital | 23 | 39,279 | 63,167 | 0.622 | 0.59 (0.37–0.88) |
| Private clinic | 46 | 67,691 | 91,611 | 0.739 | 0.68 (0.50–0.91) |
| University hospitals (national/public and private) | 6 | 5,071 | 11,527 | 0.440 | 1.18 (0.43–2.58) |
| Non-university facilities | 88 | 135,708 | 210,590 | 0.644 | 0.65 (0.52–0.80) |

**(c) Bias analysis, competing-risk scale: k × PPV (sensitivity = 1)**

| **k \ PPV** | **0.7** | **0.8** | **0.9** | **1.0** |
| --- | --- | --- | --- | --- |
| 0.0 | 0.47 | 0.54 | 0.61 | 0.67 |
| 0.5 | 0.63 | 0.72 | 0.81 | 0.90 |
| 1.0 | 0.79 | 0.90 | 1.02 | 1.13 |
| 1.5 | 0.95 | 1.09 | 1.22 | 1.36 |
| 2.0 | 1.11 | 1.27 | 1.43 | 1.58 |

**(d) Bias analysis, competing-risk scale: k × sensitivity (PPV = 1)**

| **k \ sensitivity** | **0.5** | **0.6** | **0.7** | **0.8** | **0.9** | **1.0** |
| --- | --- | --- | --- | --- | --- | --- |
| 0.0 | 1.34 | 1.12 | 0.96 | 0.84 | 0.75 | 0.67 |
| 0.5 | 1.79 | 1.50 | 1.29 | 1.13 | 1.00 | 0.90 |
| 1.0 | 2.25 | 1.87 | 1.61 | 1.41 | 1.25 | 1.13 |
| 1.5 | 2.69 | 2.25 | 1.93 | 1.69 | 1.51 | 1.36 |
| 2.0 | 3.14 | 2.62 | 2.25 | 1.98 | 1.76 | 1.58 |

**(e) Bias analysis, death-censored (net) scale: k × sensitivity (PPV = 1)**

| **k \ sensitivity** | **0.5** | **0.6** | **0.7** | **0.8** | **0.9** | **1.0** |
| --- | --- | --- | --- | --- | --- | --- |
| 0.0 | 1.58 | 1.32 | 1.13 | 0.99 | 0.88 | 0.79 |
| 0.5 | 2.11 | 1.76 | 1.51 | 1.33 | 1.18 | 1.06 |
| 1.0 | 2.64 | 2.21 | 1.90 | 1.66 | 1.48 | 1.33 |
| 1.5 | 3.17 | 2.65 | 2.28 | 1.99 | 1.77 | 1.60 |
| 2.0 | 3.70 | 3.09 | 2.65 | 2.33 | 2.07 | 1.87 |

**(f) Recording completeness and prevalence of donor history by survey year**

| **Survey year** | **Incident patients, facility survey** | **Incident patients, patient survey** | **Patient-survey coverage** | **Recorded (yes + no)** | **Recording rate** | **Donor history: yes** | **Donors per 1,000 recorded patients (95% CI)** |
| --- | --- | --- | --- | --- | --- | --- | --- |
| 2,019 | 40,885 | 38,557 | 0.943 | 23,748 | 0.616 | 15 | 0.63 (0.35–1.04) |
| 2,020 | 40,744 | 38,549 | 0.946 | 25,445 | 0.660 | 13 | 0.51 (0.27–0.87) |
| 2,021 | 40,511 | 37,968 | 0.937 | 23,727 | 0.625 | 22 | 0.93 (0.58–1.40) |
| 2,022 | 39,683 | 37,039 | 0.933 | 23,174 | 0.626 | 10 | 0.43 (0.21–0.79) |
| 2,023 | 38,764 | 36,116 | 0.932 | 23,613 | 0.654 | 20 | 0.85 (0.52–1.31) |
| 2,024 | 36,404 | 33,888 | 0.931 | 21,072 | 0.622 | 14 | 0.66 (0.36–1.11) |

**(g) Transplant-history item among patients with graft failure as primary disease**

| **Survey year** | **Graft failure, transplant item recorded** | **Transplant history: none (inconsistent)** | **Transplant history: ≥1** | **Inconsistency rate (95% CI)** |
| --- | --- | --- | --- | --- |
| 2,019 | 139 | 30 | 109 | 0.216 (0.151–0.294) |
| 2,020 | 147 | 33 | 114 | 0.224 (0.160–0.301) |
| 2,021 | 127 | 34 | 93 | 0.268 (0.193–0.354) |
| 2,022 | 134 | 24 | 110 | 0.179 (0.118–0.255) |
| 2,023 | 130 | 26 | 104 | 0.200 (0.135–0.279) |
| 2,024 | 128 | 29 | 99 | 0.227 (0.157–0.309) |
| 2019–2024 | 805 | 176 | 629 | 0.219 (0.191–0.248) |

**(h) Lower bound on item sensitivity from the transplant-society follow-up (Table S7d, e)**

| **Observed initiations in followed donors** | **Exact Poisson upper 97.5% limit** | **Minimum sensitivity, scenario B** | **Minimum sensitivity, scenario C** |
| --- | --- | --- | --- |
| 7 (reported) | 14.4 | 0.65 | 0.68 |
| 9 (reported ÷ (1 − loss to follow-up)) | 17.1 | 0.55 | 0.57 |

**(i) Joint recording of the donor-history and transplant-history items**

| **Donor-history item** | **Patients** | **Transplant item blank, n (%)** | **Transplant item unknown, n (%)** |
| --- | --- | --- | --- |
| Blank | 79,481 | 69,753 (87.8) | — |
| Recorded (yes or no) | 140,779 | 2,870 (2.0) | — |
| Unknown | 1,857 | — | 1,004 (54.1) |

**(j) Recording and prevalence of donor history by primary disease (≥1,000 patients)**

| **Primary disease (categories with ≥1 case)** | **All incident patients** | **Recorded** | **Recording rate** | **Donor history: yes** | **Donors per 1,000 recorded patients (95% CI)** |
| --- | --- | --- | --- | --- | --- |
| Diabetic nephropathy | 87,906 | 57,981 | 0.660 | 14 | 0.24 (0.13–0.41) |
| Nephrosclerosis | 40,311 | 26,397 | 0.655 | 21 | 0.80 (0.49–1.22) |
| Unknown | 31,749 | 18,101 | 0.570 | 15 | 0.83 (0.46–1.37) |
| Chronic glomerulonephritis | 31,549 | 20,169 | 0.639 | 21 | 1.04 (0.64–1.59) |
| Other | 7,857 | 4,364 | 0.555 | 15 | 3.44 (1.92–5.67) |
| Polycystic kidney disease | 5,436 | 3,707 | 0.682 | 1 | 0.27 (0.01–1.50) |
| Rapidly progressive glomerulonephritis | 3,470 | 1,919 | 0.553 | 1 | 0.52 (0.01–2.90) |
| Acute kidney injury | 2,202 | 970 | 0.441 | 1 | 1.03 (0.03–5.74) |
| Malignant hypertension | 1,632 | 1,093 | 0.670 | 0 | 0.00 (0.00–3.38) |
| Graft failure (re-initiation after transplantation) | 1,209 | 684 | 0.566 | 1 | 1.46 (0.04–8.15) |
| Renal or urinary tract tumour | 1,108 | 690 | 0.623 | 3 | 4.35 (0.90–12.71) |
| Nephritis associated with autoimmune disease | 1,081 | 656 | 0.607 | 0 | 0.00 (0.00–5.62) |

**(k) Sensitivity to the positive predictive value (PPV) of the donor-history item**

| **Assumed PPV** | **Adjusted SIR** | **Adjusted 20-y cumulative incidence, %** |
| --- | --- | --- |
| 1.00 | 1.23 | 1.13 |
| 0.90 | 1.10 | 1.02 |
| 0.85 | 1.04 | 0.96 |
| 0.80 | 0.98 | 0.90 |
| 0.75 | 0.92 | 0.85 |

**(l) Sensitivity to donor mortality (SMR relative to the general population)**

| **Assumed donor SMR** | **Death-censored donor-years 0–9 y** | **Death-censored donor-years 10–19 y** | **Expected initiations** | **Adjusted SIR** | **Unadjusted SIR** | **Adjusted 20-y cumulative incidence, %** |
| --- | --- | --- | --- | --- | --- | --- |
| 1.0 | 89,930 | 50,739 | 62.9 | 1.23 | 0.59 | 1.13 |
| 0.8 | 90,532 | 52,023 | 64.5 | 1.19 | 0.57 | 1.14 |
| 0.6 | 91,146 | 53,394 | 66.2 | 1.16 | 0.56 | 1.15 |

Notes. PPV, positive predictive value; SIR, standardized incidence ratio; SMR, standardized mortality ratio. (a) Spearman ρ (recording rate vs prevalence) 0.027, p = 0.85; Poisson rate ratio per +10% recording 1.04 (0.71–1.54); donors under uniform vs prefecture-stratified imputation 148.3 vs 147.9. Over-dispersion of prevalence: χ² = 66.6 (df 47), p = 0.031. (b) Heterogeneity of prevalence χ² = 13.6 (df 9), p = 0.14; Poisson rate ratio per +10% recording 0.93 (0.76–1.13); donors under uniform vs facility-stratified imputation 148.3 vs 151.3. Exact Poisson limits. (c–e) k, prevalence of donor history among unrecorded relative to recorded patients (k = 0, no donors among unrecorded; k = 1, missing at random, the primary estimate). Bounded range on the competing-risk scale (k 0–1, sensitivity 0.6–1, PPV 0.75–1): 0.51–1.87%; sensitivity 0.5 with k = 1: 2.25%. Values above 2% cannot be excluded; the main text reports approximately 0.5–2%. (f) Heterogeneity of prevalence across years χ² = 6.45 (df 5), p = 0.26. Missing-data factor ×1.683 = within-survey non-recording (×1.578) × facility non-response (×1.067). (g–j) (g) Clopper–Pearson limits; used as an upper anchor for the false-negative rate of history items. (h) Expected initiations ÷ sensitivity must not exceed the Poisson upper limit of the observed count. (j) Donors under uniform vs disease-stratified imputation 148.3 vs 153.3. The registry has no code for kidney failure in a solitary kidney, so true donor cases may fall in ‘other’. (k, l) (k) Adjusted cases and SIR scale linearly with the PPV; the SIR falls below 1 at a PPV of about 0.80. (l) Lower donor mortality increases death-censored donor-years, lowering the rate and the SIR and slightly raising the competing-risk cumulative incidence.

**Table S5. Characteristics of incident dialysis patients with a history of kidney donation (JRDR 2019–2024, n = 94)**

**(a) Age and prior transplantation**

| **Characteristic** | **n** |
| --- | --- |
| Age at dialysis initiation, mean (y) | 74.8 |
| <60 | 15 |
| 60–69 | 8 |
| 70–79 | 27 |
| ≥80 | 44 |
| Age at donation (2021–2024 surveys; known for 57 of 66), mean (y) | 55.7 |
| <40 | 6 |
| 40–49 | 13 |
| 50–59 | 13 |
| 60–69 | 18 |
| ≥70 | 7 |
| Prior kidney transplantation recorded | 6 |
| Primary disease incompatible with donation (polycystic kidney disease or graft failure) | 2 |

**(b) Primary disease (categories with ≥1 case)**

| **Primary disease** | **n** |
| --- | --- |
| Nephrosclerosis | 21 |
| Chronic glomerulonephritis | 21 |
| Other | 15 |
| Unknown | 15 |
| Diabetic nephropathy | 14 |
| Renal or urinary tract tumour | 3 |
| Polycystic kidney disease | 1 |
| Drug- or toxin-induced kidney injury | 1 |
| Acute kidney injury | 1 |
| Graft failure (re-initiation after transplantation) | 1 |
| Rapidly progressive glomerulonephritis | 1 |
| Virus-associated kidney disease | 0 |
| Hypertensive disorders of pregnancy | 0 |
| Congenital anomalies of the kidney and urinary tract | 0 |
| Amyloidosis | 0 |
| Other unclassifiable nephritis | 0 |
| Paraproteinaemia (myeloma) | 0 |
| Malignant hypertension | 0 |
| Chronic pyelonephritis | 0 |
| Gouty nephropathy | 0 |
| Urolithiasis | 0 |
| Renal or urinary tract tuberculosis | 0 |
| Nephritis associated with autoimmune disease | 0 |
| Hereditary disease | 0 |
| Obstructive uropathy or voiding dysfunction | 0 |
| Interstitial nephritis | 0 |

**(c) Sex by age at donation (2021–2024, known age), compared with the donor pool**

| **Age at donation, y** | **Men** | **Women** | **Women, %** | **Expected women, % (donor pool)** | **Exact binomial p** | **Men:women odds ratio vs pool (95% CI)** |
| --- | --- | --- | --- | --- | --- | --- |
| <50 | 11 | 8 | 42.1 | 63.1 | 0.093 | 2.35 (0.97–5.68) |
| 50–59 | 8 | 5 | 38.5 | 63.1 | 0.084 | 2.74 (0.94–7.95) |
| 60–69 | 13 | 5 | 27.8 | 63.1 | 0.003 | 4.45 (1.65–11.98) |
| ≥70 | 3 | 4 | 57.1 | 63.1 | 0.714 | 1.28 (0.32–5.12) |
| <60 (pooled) | 19 | 13 | 40.6 | 63.1 | 0.010 | 2.50 (1.25–5.00) |
| ≥60 (pooled) | 16 | 9 | 36.0 | 63.1 | 0.007 | 3.04 (1.37–6.74) |
| All (known age) | 35 | 22 | 38.6 | 63.1 | <0.001 | 2.72 (1.61–4.61) |

Notes. (a) Means approximated from age-band midpoints. (c) Age at donation unknown: men 8, women 1. Expected female share assumes independence of sex and age in the donor pool (63.1%, donor-year-weighted), as JSCRT reports only marginal distributions; no sex–age distribution consistent with the overall female share yields an expected share as low as the observed 38.6%.

**Table S6. Living kidney donors in Japan by year of donation (denominator)**

| **Year of donation** | **Living-donor transplants** | **Age <40, proportion** | **40–49** | **50–59** | **60–69** | **≥70** | **Women, %** | **Source of age distribution** |
| --- | --- | --- | --- | --- | --- | --- | --- | --- |
| 1,999 | 566 | 0.117 | 0.221 | 0.362 | 0.237 | 0.064 | — | interpolated |
| 2,000 | 603 | 0.117 | 0.221 | 0.362 | 0.237 | 0.064 | 63.5 | JSCRT report |
| 2,001 | 554 | 0.113 | 0.203 | 0.364 | 0.248 | 0.071 | — | interpolated |
| 2,002 | 637 | 0.109 | 0.186 | 0.366 | 0.260 | 0.079 | 62.3 | JSCRT report |
| 2,003 | 728 | 0.129 | 0.187 | 0.378 | 0.251 | 0.055 | 60.0 | JSCRT report |
| 2,004 | 731 | 0.130 | 0.207 | 0.367 | 0.225 | 0.071 | 62.5 | JSCRT report |
| 2,005 | 835 | 0.130 | 0.176 | 0.356 | 0.257 | 0.080 | 61.6 | JSCRT report |
| 2,006 | 941 | 0.124 | 0.187 | 0.348 | 0.253 | 0.089 | 60.6 | JSCRT report |
| 2,007 | 1,043 | 0.109 | 0.172 | 0.344 | 0.300 | 0.075 | 60.9 | JSCRT report |
| 2,008 | 994 | 0.097 | 0.175 | 0.352 | 0.287 | 0.089 | 63.7 | JSCRT report |
| 2,009 | 1,122 | 0.099 | 0.172 | 0.338 | 0.317 | 0.074 | 63.8 | JSCRT report |
| 2,010 | 1,277 | 0.074 | 0.172 | 0.306 | 0.361 | 0.086 | 61.5 | JSCRT report |
| 2,011 | 1,386 | 0.078 | 0.156 | 0.294 | 0.389 | 0.082 | 62.7 | JSCRT report |
| 2,012 | 1,420 | 0.077 | 0.162 | 0.296 | 0.340 | 0.124 | 63.3 | JSCRT report |
| 2,013 | 1,438 | 0.068 | 0.176 | 0.289 | 0.365 | 0.102 | 61.0 | JSCRT report |
| 2,014 | 1,479 | 0.069 | 0.177 | 0.296 | 0.340 | 0.117 | 62.9 | JSCRT report |
| 2,015 | 1,503 | 0.057 | 0.164 | 0.273 | 0.375 | 0.131 | 64.8 | JSCRT report |
| 2,016 | 1,471 | 0.070 | 0.178 | 0.273 | 0.353 | 0.127 | 63.8 | JSCRT report |
| 2,017 | 1,544 | 0.051 | 0.164 | 0.269 | 0.371 | 0.146 | 63.5 | JSCRT report |
| 2,018 | 1,683 | 0.058 | 0.177 | 0.297 | 0.308 | 0.160 | 62.8 | JSCRT report |
| 2,019 | 1,827 | 0.037 | 0.161 | 0.301 | 0.337 | 0.164 | 62.3 | JSCRT report |
| 2,020 | 1,569 | 0.053 | 0.141 | 0.275 | 0.356 | 0.176 | 67.1 | JSCRT report |
| 2,021 | 1,649 | 0.042 | 0.158 | 0.294 | 0.338 | 0.169 | 65.2 | JSCRT report |
| 2,022 | 1,594 | 0.046 | 0.146 | 0.313 | 0.314 | 0.182 | 64.0 | JSCRT report |
| 2,023 | 1,753 | 0.049 | 0.148 | 0.320 | 0.295 | 0.188 | 63.1 | JSCRT report |
| 2,024 | 1,668 | 0.034 | 0.152 | 0.313 | 0.307 | 0.194 | 64.2 | JSCRT report (Ishoku 61:127) |

Notes. JSCRT, Japanese Society for Clinical Renal Transplantation. Annual counts: Japan Society for Transplantation Fact Book. Age distribution and female share: JSCRT annual reports; 1999 carried from 2000 and 2001 interpolated (together 2.9% of donor-years in the 10–19-y interval; replacing them changed the 20-y net risk by ≤0.003 percentage points).

**Table S7. Validity of the period estimate: donation-cohort check, prevalent-patient check and external comparison with the transplant-society donor follow-up**

**(a) Initiations within 20 years by reconstructed donation cohort: observed vs expected**

| **Donation cohort** | **Observed** | **Expected** | **O/E (95% CI)** | **Donor-years 0–9 y** | **Donor-years 10–19 y** |
| --- | --- | --- | --- | --- | --- |
| 1999–2004 | 4 | 5.6 | 0.71 (0.19–1.82) | 0 | 12,098 |
| 2005–2009 | 15 | 13.5 | 1.11 (0.62–1.83) | 561 | 29,049 |
| 2010–2014 | 13 | 10.6 | 1.23 (0.65–2.10) | 24,956 | 17,044 |
| 2015–2019 | 5 | 5.1 | 0.98 (0.32–2.30) | 47,254 | 0 |
| 2020–2024 | 0 | 2.2 | 0.00 (0.00–1.69) | 20,280 | 0 |

**(b) Prevalent patients with donor history (31 December 2024) by age and vintage: observed vs expected**

| **Age at survey, y** | **Dialysis vintage, y** | **Donors observed** | **Non-donors in this vintage, %** | **Donors expected** | **O/E (95% CI)** |
| --- | --- | --- | --- | --- | --- |
| <65 | 0–3 y | 14 | 34.5 | 6.6 | 2.13 (1.17–3.58) |
| <65 | 4–9 y | 3 | 34.4 | 6.5 | 0.46 (0.09–1.34) |
| <65 | 10–19 y | 2 | 21.4 | 4.1 | 0.49 (0.06–1.77) |
| <65 | ≥20 y | 0 | 9.6 | 1.8 | 0.00 (0.00–2.02) |
| 65–74 | 0–3 y | 14 | 33.4 | 7.7 | 1.82 (1.00–3.06) |
| 65–74 | 4–9 y | 4 | 33.0 | 7.6 | 0.53 (0.14–1.35) |
| 65–74 | 10–19 y | 4 | 22.3 | 5.1 | 0.78 (0.21–2.00) |
| 65–74 | ≥20 y | 1 | 11.4 | 2.6 | 0.38 (0.01–2.12) |
| 75–84 | 0–3 y | 28 | 41.3 | 18.6 | 1.51 (1.00–2.18) |
| 75–84 | 4–9 y | 10 | 33.6 | 15.1 | 0.66 (0.32–1.21) |
| 75–84 | 10–19 y | 4 | 17.6 | 7.9 | 0.50 (0.14–1.29) |
| 75–84 | ≥20 y | 3 | 7.4 | 3.3 | 0.90 (0.19–2.63) |
| ≥85 | 0–3 y | 17 | 48.4 | 15.5 | 1.10 (0.64–1.76) |
| ≥85 | 4–9 y | 14 | 37.0 | 11.8 | 1.18 (0.65–1.99) |
| ≥85 | 10–19 y | 1 | 12.3 | 3.9 | 0.25 (0.01–1.41) |
| ≥85 | ≥20 y | 0 | 2.3 | 0.7 | 0.00 (0.00–4.97) |

**(c) As (b), pooled across age bands**

| **Dialysis vintage, y** | **Donors observed** | **Donors expected** | **O/E (95% CI)** |
| --- | --- | --- | --- |
| 0–3 y | 73 | 48.3 | 1.51 (1.18–1.90) |
| 4–9 y | 31 | 41.1 | 0.75 (0.51–1.07) |
| 10–19 y | 11 | 21.1 | 0.52 (0.26–0.93) |
| ≥20 y | 4 | 8.5 | 0.47 (0.13–1.20) |

**(d) Transplant-society donor follow-up (donations 2009–2022): reported data**

| **Item** | **Value** |
| --- | --- |
| Donor-years with known dialysis status (yearly time points 1–11 y) | 40,261 |
| Donor-years entered (any status) | 53,611 |
| Reported dialysis initiations (cumulative; upper bound of unique donors) | 7 |
| Reported rate per 10,000 donor-years (95% CI) | 1.74 (0.70–3.58) |

**(e) Transplant-society follow-up: reported vs expected under the period rates of this study**

|  | **Unadjusted (A)** | **Adjusted (B)** | **Adjusted, death-censored (C)** |
| --- | --- | --- | --- |
| Expected initiations | 4.5 | 9.4 | 9.8 |
| Observed / expected (95% CI) | 1.55 (0.62–3.19) | 0.74 (0.30–1.53) | 0.71 (0.29–1.47) |
| Observed / expected after allocating unreported status at the same rate (95% CI) | 2.06 (0.96–3.87) | 0.99 (0.46–1.86) | 0.95 (0.44–1.78) |

Notes. O/E, observed/expected with exact Poisson limits on the observed count. (a) Expected under the unadjusted period rates. Heterogeneity χ² = 3.35 (df 4), p = 0.50; trend per 5 later donation years 0.99 (0.73–1.34). (b, c) Expected from the vintage distribution of patients without donor history in the same age band; χ² = 22.3 (df 3), p < 0.001. In the 2019 year-end survey 47 of 181 recorded donors (26%) also had a recorded transplant, versus 6 of 94 (6%) in the incident surveys. (d, e) Loss to follow-up at 10 y 26%; status unknown among entered donors 25%. Reported initiations may count the same donor at several time points (upper bound). Allocating unreported status at the same rate applies the assumption under test and is not independent evidence.
